# Hay Fever

**Published:** 1873-02

**Authors:** 


### HAY FEVER.

Take a saturated solution of sulphate of quinine, lie on the back, let a few drops fall into each nostril, and turn the head from side to side, so as to cause the mixture to run down towards the throat. Relief is almost instantaneous. In mild cases, a solution less strong, put into the hollow of the hand, and snuffed up the nose so as to carry it back into the throat and mouth, may answer; especially in that form of the disease which follows the presence of a living thing, called vibrio, in the recesses of the nostrils; for these are only dislodged by a violent sneeze, not being found in the water which runs from the nose. Hay fever is alleviated, and sometimes prevented, by a removal to a different atmosphere; but medicines, judiciously administered, have been successful, as it arises from a disordered stomach and liver.

